# Validating superior maize hybrids in all India coordinated trials using REMATTOOL-R: a decision support approach

**DOI:** 10.3389/fpls.2026.1885434

**Published:** 2026-07-07

**Authors:** Sunil Neelam, Jyothi Bhoga, Jyostna Bellamkonda, Bhadru Dharavath, Mukesh Choudhary, Pardeep Kumar, Ramesh Kumar Phagna, Shyam Bir Singh, Dhandapani Appavoo, Hanuman Sahay Jat

**Affiliations:** 1 Winter Nursery Centre, ICAR- Indian Council of Agricultural Research, Indian Institute of Maize Research, Hyderabad, India; 2 Acharya N. G. Ranga Agricultural University, Bapatla, India; 3 Maize Research Centre, Professor Jayashankar Telangana State Agricultural University, Hyderabad, India; 4 ICAR- Indian Council of Agricultural Research, Indian Institute of Maize Research, Ludhiana, India; 5 ICAR- Indian Council of Agricultural Research, National Academy of Agricultural Research Management, Hyderabad, India

**Keywords:** AICRP maize, coordinated breeding programmes, decision-support tool, maize hybrid advancement, multi-environment trials, REMATTOOL-R

## Abstract

**Introduction:**

The identification and advancement of superior maize hybrids under the All India Coordinated Research Project (AICRP) on Maize rely on multi-environment evaluation integrating grain yield, maturity, and agronomic performance. Interpretation of large multi-environment datasets is often complex, time-consuming, and susceptible to subjectivity, highlighting the need for objective and reproducible decision-support tools. This study evaluated the effectiveness of REMATTOOL-R (Relative Maturity Adjustment Tool in R) in validating the existing hybrid advancement framework adopted under the AICRP on Maize.

**Methods:**

Multi-environment trial data from the National Initial Varietal Trial (NIVT)-Late conducted during Kharif 2020–21 across five locations representing the Central West Zone (CWZ) of India were analysed. The dataset comprised 45 entries, including 40 experimental hybrids, four commercial checks, and one filler entry. REMATTOOL-R integrated grain yield with days to 50% anthesis, grain moisture at harvest, and harvested plant stand to facilitate simultaneous evaluation of grain yield, maturity, and adaptation-related traits. Least-square means generated from mixed-model analysis were used to identify superior hybrids based on a predefined grain yield superiority threshold (≥5%) over the standard check while maintaining comparable maturity and agronomic performance.

**Results:**

REMATTOOL-R enabled rapid visualization and integrated assessment of multiple agronomic traits, allowing objective identification of superior hybrids. Five experimental hybrids—PM 21109L (Entry 30), R8050 (Entry 35), PM 21111L (Entry 32), BIO 978 (Entry 4), and DKC 9226 (Entry 9)—recorded ≥5% higher grain yield than the standard check Bio 9682 while maintaining statistically comparable days to 50% anthesis, grain moisture at harvest, and harvested plant stand. All five hybrids identified by REMATTOOL-R corresponded with the official AICRP decisions for advancement from NIVT to Advanced Varietal Trial-I (AVT-I), while three hybrids (R8050, PM 21111L, and DKC 9226) progressed further to AVT-II during subsequent testing cycles, confirming the reliability of the analytical framework.

**Discussion:**

The findings demonstrate that REMATTOOL-R provides an efficient, transparent, and reproducible framework for the simultaneous evaluation of grain yield, maturity, and adaptation-related traits in maize multi-environment trials. By complementing the existing AICRP hybrid evaluation procedure, the tool facilitates objective advancement decisions and reduces subjectivity associated with manual interpretation of complex datasets. REMATTOOL-R therefore represents a valuable decision-support approach for coordinated maize breeding programmes and has considerable potential for application in large-scale hybrid evaluation systems.

## Introduction

Maize (*Zea mays* L.) is one of the most important cereal crops globally and contributes substantially to food, feed, fodder and industrial sectors because of its high productivity, wider adaptability and diverse end uses. Owing to its nutritional and economic importance, maize is often regarded as a “golden cereal crop” contributing significantly to global food and nutritional security (Kaushal et al., 2023). Globally, maize is cultivated on more than 200 million hectares with annual production exceeding 1.2 billion tonnes (FAOSTAT, 2023). In India, maize has emerged as one of the fastest-growing cereal crops owing to the increasing demand from poultry feed industries, starch processing sectors, food products and ethanol blending programmes (Yadav et al., 2016). According to *Agricultural Statistics at a Glance 2024*, maize is cultivated on nearly 12 million hectares with steadily increasing production and productivity levels (Ministry of Agriculture & Farmers Welfare, 2024).

Despite substantial progress in maize improvement, productivity in many regions remains constrained by major biotic and abiotic stresses including drought, heat stress, diseases and climatic variability (Akasairi Ocwa et al., 2023; Njeru et al., 2023). Environmental fluctuations associated with climate change have further intensified the challenge of developing stable and high-yielding hybrids adapted to diverse agro-ecological conditions (IPCC, 2014). Consequently, identification of productive hybrids possessing stable performance across environments has become a major objective of modern maize breeding programmes (Cooper et al., 2014).

To identify suitable hybrids for commercial cultivation, the All India Coordinated Research Project (AICRP) on Maize follows a rigorous multi-location testing system in which experimental entries are evaluated across diverse agro-climatic regions before advancement and release. Multi-environment trials (METs) constitute the foundation of coordinated varietal assessment because they facilitate evaluation of genotype performance across contrasting environments and enable identification of hybrids with broad or specific adaptation (Yan and Tinker, 2006). Under the AICRP testing framework, hybrids are promoted sequentially from National Initial Varietal Trials (NIVT) to Advanced Varietal Trial-I (AVT-I) and subsequently to AVT-II based on standardized criteria involving grain yield superiority, statistical significance and adaptation-related parameters (AICRP-Maize, 2023).

In maize breeding programmes, grain yield is evaluated together with important agronomic and phenological traits including days to 50% anthesis, grain moisture at harvest, harvested plant stand and disease resistance. Since grain yield is strongly influenced by crop duration and environmental variability, simultaneous assessment of productivity and maturity behaviour is essential for identifying superior hybrids (Bänziger et al., 2000). This becomes particularly important under stress-prone and rainfed environments where prolonged maturity may expose hybrids to terminal drought stress and reduce adaptation and yield stability (Cooper et al., 2014). Previous studies have also highlighted the importance of yield-contributing traits and their association with grain yield improvement in maize. Under the AICRP testing system, days to 50% anthesis and grain moisture content are also considered important maturity-related parameters influencing hybrid advancement across environments (AICRP-Maize, 2023).

Although multi-location trials generate reliable information on genotype performance, interpretation of large datasets involving multiple agronomic traits across locations and seasons remains a major challenge in breeding programmes. Breeders and varietal evaluation committees often examine extensive statistical summaries, comparative tables and graphical outputs before making advancement decisions. Such manual interpretation may increase the possibility of computational inconsistencies and subjective bias, particularly when large numbers of entries are evaluated simultaneously across environments and maturity groups (Yan, 2014). Several analytical approaches including AMMI, GGE biplot analysis, META-R, Fieldbook and SAS have been widely used for genotype × environment interaction analysis and management of breeding datasets (Alvarado et al., 2020; Gauch, 2006; SAS Institute Inc., 2011; Vivek et al., 2007). However, effective interpretation still requires simultaneous consideration of grain yield together with maturity-related and agronomic traits to support balanced advancement decisions.

To improve combined interpretation of these traits, REMATTOOL-R (Relative Maturity Adjustment Tool in R) was developed as a breeder-oriented analytical approach for evaluation of multi-location trial data. The tool integrates grain yield with important agronomic and phenological traits including anthesis date, grain moisture content and harvested plant stand, enabling simultaneous assessment of productivity and maturity behaviour across environments (Machida et al., 2023). REMATTOOL-R generates comparative visual outputs relative to standard check hybrids and facilitates identification of entries combining superior grain yield with desirable maturity characteristics. Such approaches improve transparency, reproducibility and efficiency in hybrid selection workflows and support objective decision-making in coordinated breeding programmes.

The present investigation was undertaken to validate the hybrid promotion system followed under AICRP on Maize through simultaneous evaluation of grain yield and key agronomic traits using REMATTOOL-R. The study aimed to examine whether hybrids identified through the established AICRP promotion procedure correspond with those selected through trait-based analysis of multi-location trial data. Particular emphasis was placed on assessing the consistency and reliability of advancement decisions and evaluating the usefulness of REMATTOOL-R as a complementary framework for transparent and reproducible hybrid selection.

## Materials and methods

### Implementation, program description and requirements

The analytical workflow in the present study was implemented using REMATTOOL-R, an R-based decision-support tool with a graphical user interface (GUI) designed for identifying superior maize hybrids from multi-environment trial data. The software operates within the R statistical computing environment and is compatible with both R (R Core Team, 2021) and R Studio (R Studio Team, 2020). R software can be downloaded from the Comprehensive R Archive Network (CRAN) and R Studio from the official Posit/R Studio website according to the user’s operating system requirements. Detailed installation and configuration procedures for REMATTOOL-R are described by Machida et al. (2023).

### Dataset used in REMATTOOL-R

The dataset used for analysis was obtained from the National Initial Variety Trial (NIVT) Late conducted under the All India Coordinated Research Project (AICRP) on Maize during the Kharif 2020–21 season. The trial evaluated 45 entries, comprising 40 experimental hybrids, four commercial checks (Bio 9682, CMH 08-282, CMH 08-287, and NK 6240), and one filler entry (DHM 117). The experiment was conducted across five locations representing Zone 5 (Central West Zone, CWZ), namely Ambikapur, Banswara, Chhindwara, Godhra, and Udaipur.

The field experiments were laid out in a randomized block design (RBD) with three replications at each location. Each experimental plot consisted of two rows of 4 m length, maintaining a spacing of 60 cm between rows and 20 cm between plants within rows. Standard agronomic and crop management practices recommended for the respective regions were followed uniformly across locations.

Data were recorded for grain yield (plot basis), days to 50% anthesis, grain moisture at harvest, and harvested plant stand. The trait data obtained from individual environments were subjected to statistical analysis within the AICRP evaluation framework, and least square means (LS means) across environments were generated for each genotype. These LS means were subsequently used as input for REMATTOOL-R analysis.

The REMATTOOL-R workflow involved sequential data importation, trait standardization, superiority threshold evaluation relative to check hybrids, maturity adjustment based on anthesis duration, and final identification of superior hybrids using multi-trait selection criteria. The analytical framework accounted for environmental variation and genotype × environment interaction effects through summarized multi-environment LS mean estimates.

Variance components were estimated using restricted maximum likelihood (REML) procedures within the mixed model framework. Genotype × environment interaction effects were incorporated to account for differential hybrid responses across testing locations. Statistical significance was assessed at the 5% probability level (P < 0.05). Model assumptions including normality of residuals, homogeneity of variance, and independence of residual errors were evaluated through standard diagnostic procedures.

The general statistical model underlying the multi-environment analysis can be represented as:


$$Y_{ijk}=\mu +G_{i}+E_{j}+{\left(GE\right)}_{ij}+R_{k(j}+{Є}_{ijk}$$

where:

$Y_{ijk}$ = observed value of the trait for the i^th^ genotype in the j^th^ environment and k^th^ replication

μ = overall mean

$G_{i}\;$ = effect of the i^th^ genotype

$E_{j}$ = effect of the j^th^ environment

${\left(GE\right)}_{ij}$ = interaction effect between the i^th^ genotype and the j^th^ environment

$R_{k(j}$ = effect of the k^th^ replication nested within the j^th^ environment

${Є}_{ijk}$= residual experimental error associated with the observation $Y_{ijk}$

The final superior entries were identified based on integrated multi-trait performance considering grain yield, flowering duration, grain moisture, and harvested plant stand simultaneously within the REMATTOOL-R framework.

**Figure 1:**
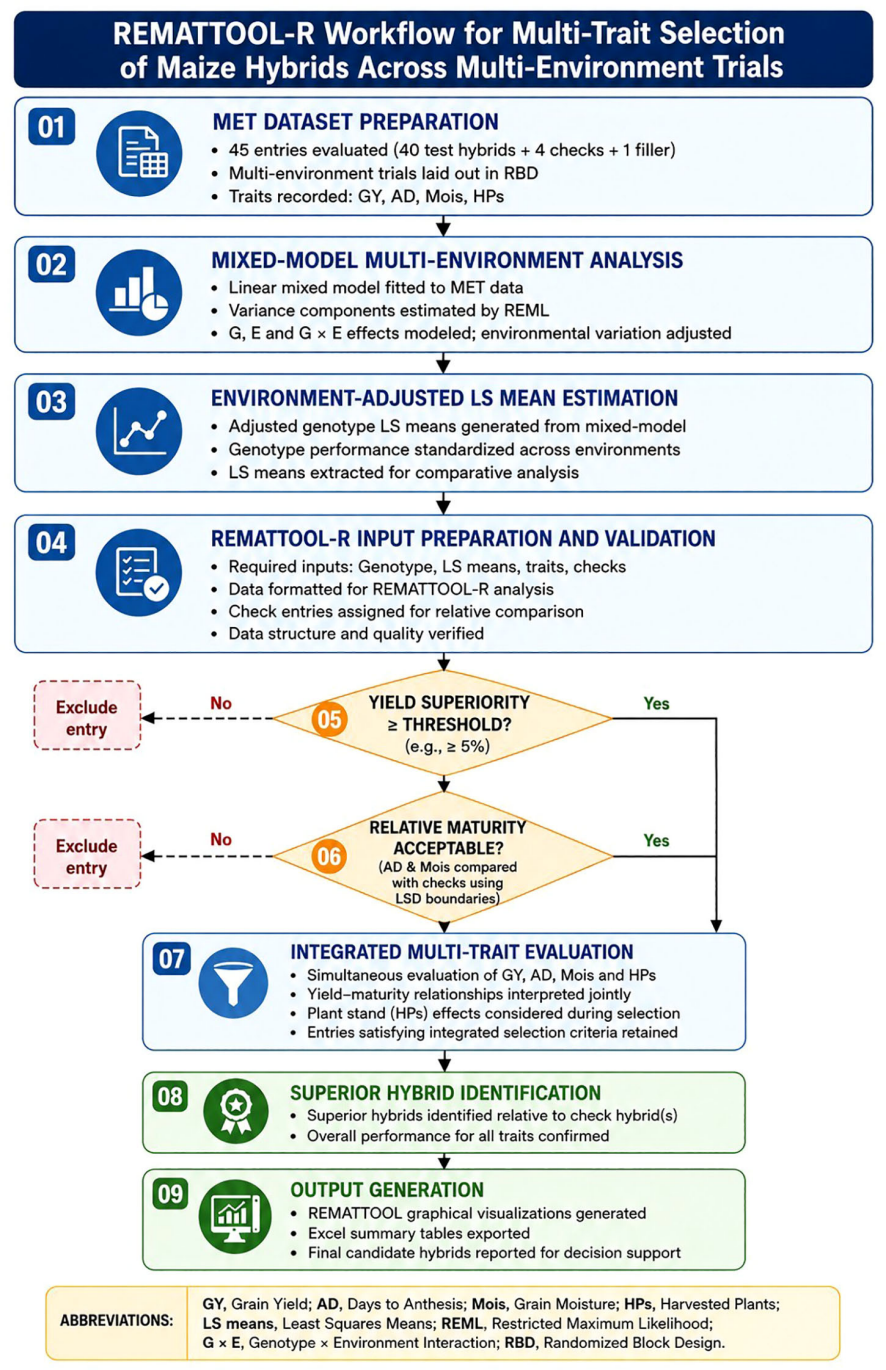
Workflow of the REMATTOOL-R analytical framework for multi-trait evaluation of maize hybrids across multi-environment trials. The workflow includes dataset preparation, mixed-model analysis, least-square (LS) mean estimation, REMATTOOL-R input preparation, evaluation of grain yield superiority relative to the standard check, integrated assessment of days to 50% anthesis, grain moisture at harvest, and harvested plant stand, followed by identification of superior hybrids and generation of graphical and tabulated outputs.

The analytical workflow followed in the present study is summarized in **Figure 1**. The workflow includes dataset preparation, mixed-model multi-environment analysis, LS mean estimation, REMATTOOL-R input preparation, multi-trait screening relative to check hybrids, and final identification of superior hybrids.

## Results

REMATTOOL-R generated graphical and tabulated outputs for simultaneous evaluation of grain yield, days to 50% anthesis, grain moisture content, and harvested plant stand relative to check hybrids. The graphical outputs included individual x–y scatterplots representing key agronomic relationships together with integrated correlation analyses among evaluated traits. In parallel, Excel outputs provided trait-based summaries and multi-trait filtering combinations for rapid identification of superior entries satisfying predefined selection criteria.

### Graph outputs

#### Yield vs. days to 50% anthesis (X–Y graph)

**Figure 2:**
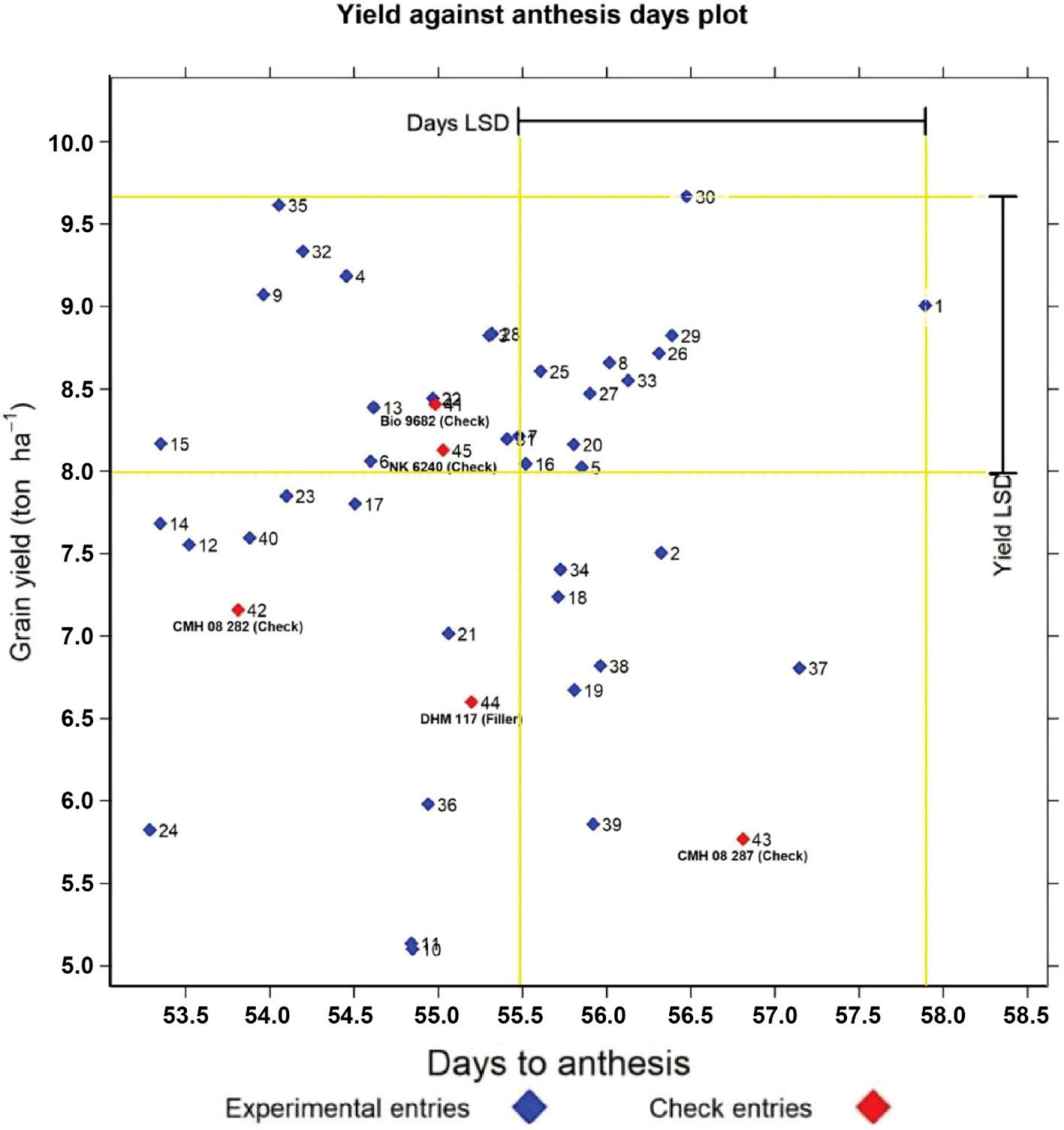
Relationship between grain yield (t ha⁻¹) and days to 50% anthesis for experimental and check maize hybrids generated using REMATTOOL-R. Vertical and horizontal dashed lines represent the least significant difference (LSD) thresholds for anthesis and grain yield, respectively, facilitating comparison of experimental entries with the standard check Bio 9682.

The REMATTOOL-R analysis generated an x–y scatter plot illustrating the relationship between grain yield (t ha^-1^) and days to 50% anthesis (**Figure 2**). The graph includes experimental entries (blue diamonds) and check hybrids (red diamonds). Vertical and horizontal dashed lines indicate the least significant differences (LSD) for anthesis days and grain yield, respectively. These thresholds facilitate classification of entries as statistically similar (S) or not significantly different but numerically higher (NSHigh) relative to the check hybrid.

The best check (standard control), Bio 9682 (Entry 41), recorded a grain yield of approximately 8.4 t ha^-^¹ and an anthesis duration of about 55 days. Entries positioned within the anthesis LSD interval of Bio 9682 and above the grain yield threshold are considered of primary interest. Entry 30 exhibited the highest grain yield (~9.7 t ha^-^¹) and remained within the anthesis LSD range of Bio 9682, indicating statistical comparability in flowering duration (CodeAD = NSHigh). However, because its anthesis duration was numerically higher than the check, its advancement should be interpreted with caution, as repeated selection of such entries could gradually shift maturity duration within the maturity group.

Entry 35 (~9.6 t ha^-^¹) combined high grain yield with anthesis duration comparable to the check. Similarly, entries 32, 9, and 4 exceeded Bio 9682 in grain yield while maintaining anthesis values within the LSD threshold. Overall, entries 30, 35, 32, 9, and 4 demonstrated superior yield performance without statistically significant differences in anthesis duration relative to Bio 9682, making them suitable candidates for advancement, subject to maturity considerations.

#### Yield vs. grain moisture at harvest (X–Y graph)

**Figure 3:**
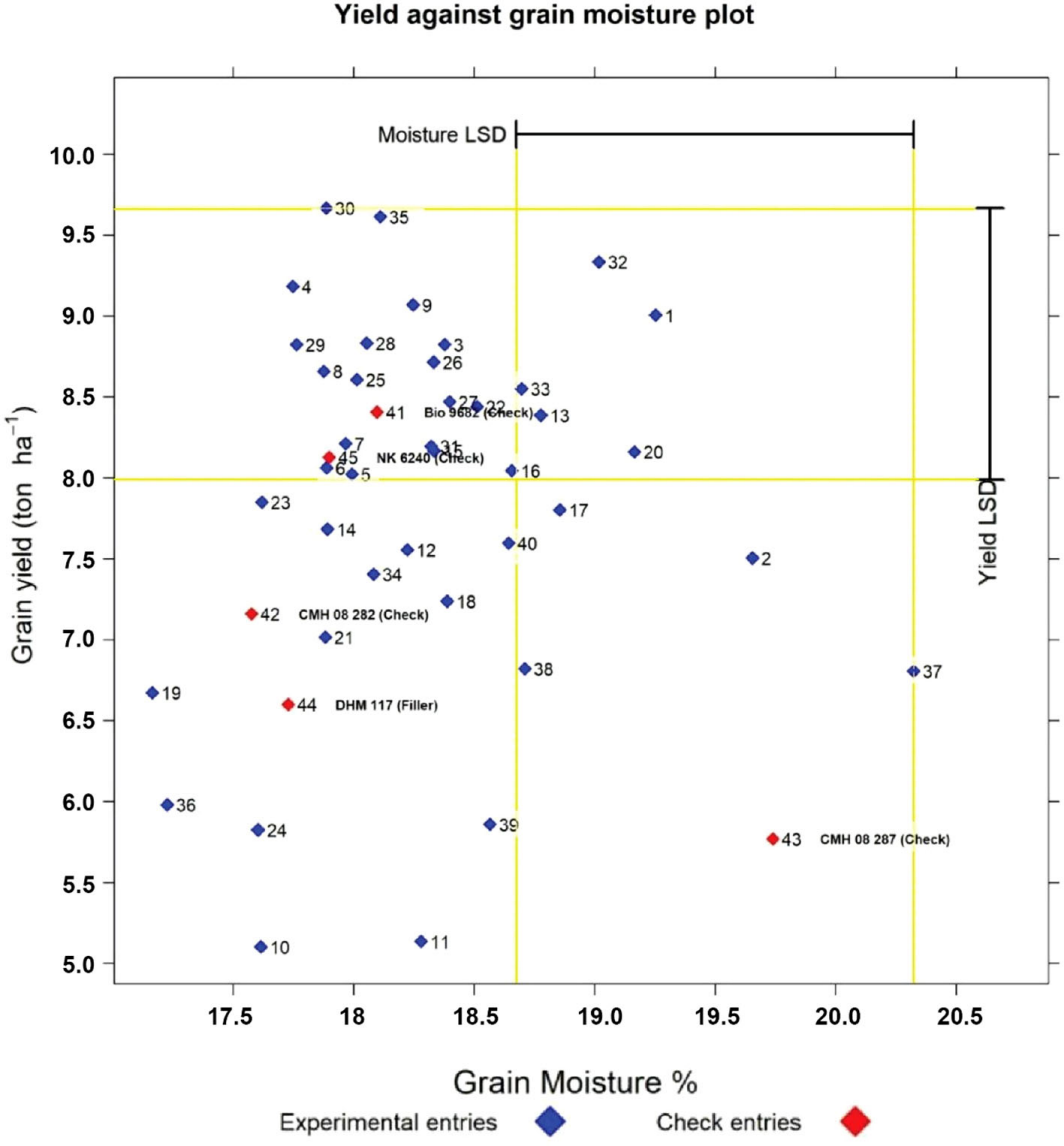
Relationship between grain yield (t ha⁻¹) and grain moisture at harvest (%) for experimental and check maize hybrids generated using REMATTOOL-R. Vertical and horizontal dashed lines represent the least significant difference (LSD) thresholds used to compare experimental entries with the standard check Bio 9682.

The Yield vs. Grain Moisture plot (**Figure 3**) evaluated the performance of experimental entries relative to the check hybrid Bio 9682 (Entry 41), which recorded a grain yield of 8.4 t ha^-^¹ and grain moisture content of 18.1% at harvest. LSD boundaries for grain yield and moisture content defined the limits of statistical equivalence between entries and the check.

All evaluated entries exhibited grain moisture values below the 26% physiological maturity threshold recommended for harvest. Although later harvesting may enhance discrimination among maturity classes, the observed moisture values provided a reliable indicator of relative maturity. The graphical output enabled rapid identification of entries combining grain moisture comparable to the check with superior yield performance.

Entries PM 21109L (30), R8050 (35), PM 21111L (32), BIO 978 (4), and DKC 9226 (9) recorded ≥5% higher grain yield than Bio 9682 while maintaining grain moisture values that were statistically comparable to the check (CodeMois = S or NSHigh). Their placement within the LSD boundaries indicates yield improvement without major changes in physiological maturity. In contrast, ADV 7211 (Entry 1) achieved high yield but displayed elevated grain moisture, suggesting relatively later maturity and potentially higher drying requirements after harvest. Entries 43 and 37 performed poorly for both yield and grain moisture and were therefore not considered suitable for advancement. Some entries (e.g., 25, 26, and 27) exhibited grain moisture comparable to the check but failed to satisfy the minimum yield threshold.

#### Yield vs. number of harvested plants (X–Y graph)

**Figure 4:**
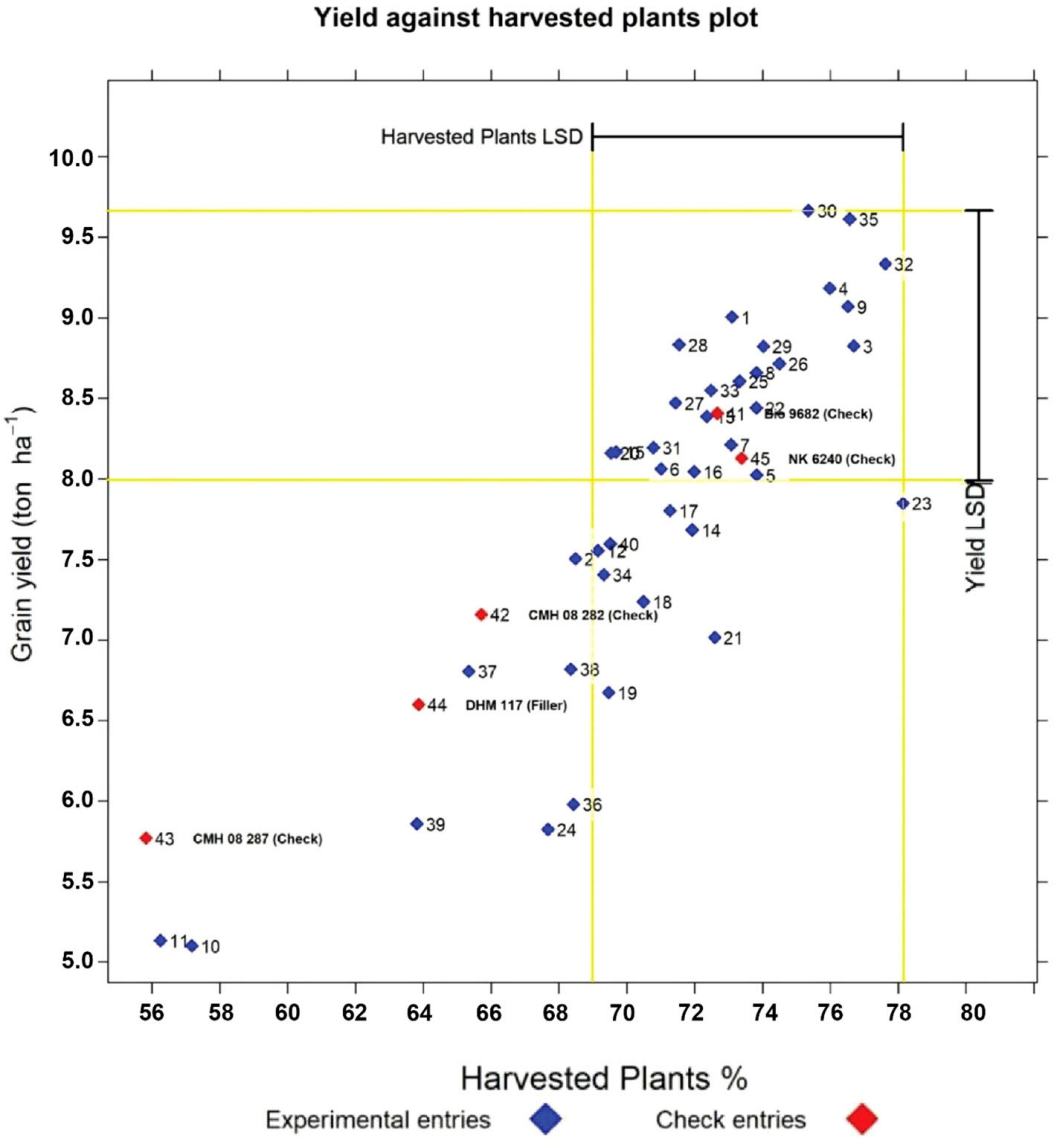
Relationship between grain yield (t ha⁻¹) and harvested plant stand (HPs) for experimental and check maize hybrids generated using REMATTOOL-R. Vertical and horizontal dashed lines represent the least significant difference (LSD) thresholds used to compare experimental entries with the standard check Bio 9682.

The grain yield versus harvested plant stand plot (**Figure 4**) provides insight into whether yield comparisons among entries were influenced by differences in plant establishment. Ideally, all hybrids should possess similar plant stand levels at harvest to permit fair comparison of genetic yield potential.

Bio 9682 (Entry 41) recorded a grain yield of 8.4 t ha^-^¹ with an average harvested plant stand of approximately 36.7 plants per plot. The graph revealed a general trend in which higher-yielding entries tended to exhibit higher harvested plant counts, suggesting some influence of stand establishment on productivity. LSD boundaries for yield and harvested plants were used to identify entries exhibiting genuine yield gains without significant differences in plant stand.

Five entries—PM 21109L (30), R8050 (35), PM 21111L (32), BIO 978 (4), and DKC 9226 (9)—met these criteria, combining ≥5% yield superiority with harvested plant counts that were statistically comparable to the check. ADV 7211 (Entry 1) also exhibited high yield with harvested plant counts within the LSD threshold, indicating that its performance was not substantially confounded by stand variation. Conversely, entries 43, 44, 39, and 10 recorded both low yield and poor harvested plant stands, whereas entries 12, 18, and 21 did not achieve sufficient yield advantage despite acceptable plant counts. These findings indicate that entries 30, 35, 32, 4, and 9 demonstrated meaningful yield improvement attributable primarily to genetic performance rather than differences in plant density.

#### Pairwise correlation matrix of agronomic traits

**Figure 5:**
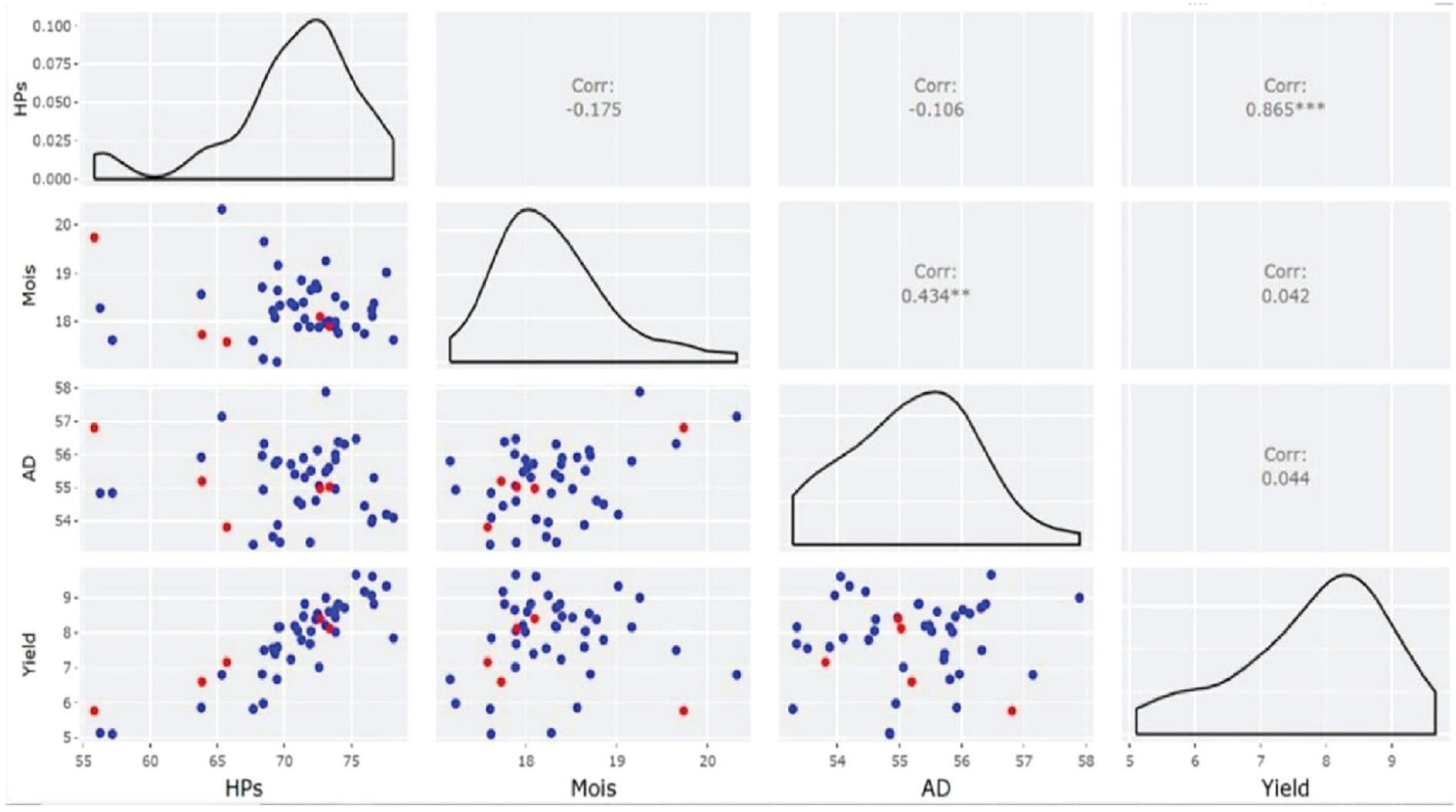
Pairwise correlation matrix illustrating the relationships among harvested plant stand (HPs), grain moisture at harvest (Mois), days to 50% anthesis (AD), and grain yield (Yield) for maize hybrids evaluated using the REMATTOOL-R framework.

A pairwise correlation matrix was constructed to examine relationships among harvested plants (HPs), grain moisture content at harvest (Mois), days to 50% anthesis (AD), and grain yield (t ha^-^¹) (**Figure 5**). The matrix included scatter plots (lower triangle), trait distributions (diagonal panels), and Pearson correlation coefficients (upper triangle). Experimental entries were represented in blue and check entries in red.

Grain yield exhibited a strong positive correlation with harvested plants (r = 0.854, P < 0.001), highlighting the importance of adequate plant stand in determining yield performance. The strong positive association between grain yield and harvested plants further suggests that harvested plant stand may be considered as a covariate during yield analysis when stand variation is substantial. This result supports the REMATTOOL-R approach of evaluating yield gains in conjunction with plant stand to minimize confounding effects arising from stand variation.

A moderate positive correlation was observed between grain moisture and anthesis duration (r = 0.434, P *<* 0.01), indicating that later-flowering entries tended to retain slightly higher grain moisture at harvest. This relationship is consistent with the physiological association between flowering time and grain dry-down behavior. Other correlations were weak and non-significant, including yield versus grain moisture (r = 0.044) and yield versus anthesis duration (r = 0.043), suggesting relative independence among these traits within the evaluated dataset. Harvested plants also showed no significant association with grain moisture (r = −0.121) or anthesis duration (r = −0.088), indicating that variation in plant stand did not substantially bias maturity-related traits.

#### Excel output options for identifying superior experimental entries

In addition to graphical visualizations, REMATTOOL-R generated Excel outputs corresponding to each designated check hybrid. These outputs included single-trait comparisons (Yield vs. AD, Yield vs. Mois, Yield vs. HPs) and integrated multi-trait combinations involving anthesis duration, grain moisture content, and harvested plant stand. The Excel summaries enabled rapid identification and ranking of entries satisfying predefined yield superiority and maturity-related selection criteria relative to the check hybrid.

#### Yield versus days to 50% anthesis (yield vs. AD)

**Tables 1**–**7** summarize experimental entries evaluated relative to the commercial check Bio 9682. **Table 1** presents the performance of selected hybrids for grain yield and days to 50% anthesis (AD). Bio 9682 recorded a grain yield of 8.41 t ha^-1^ with an AD value of 55 days.

**Table 1:** Output for the check variety Bio 9682 in the “yield vs. AD”.

Name	Entry	Pedigree	Yield	AD	Percentage yield	CodeAD
Bio 9682 (Check)	41	Bio 9682 (Check)	8.4	55.0	0	S
PM 21109L	30	Ped_30	9.7	56.5	15.0	NSHigh
R8050	35	Ped_35	9.6	54.1	14.3	S
PM 21111L	32	Ped_32	9.3	54.2	10.9	S
BIO 978	4	Ped_04	9.2	54.5	9.2	S
DKC 9226	9	Ped_09	9.1	54.0	7.8	S

AD, Days to 50% anthesis; PercentageYield, Percentage yield above the check (Bio 9682); CodeAD, Code for Days to 50% anthesis; S, Similar and not significantly different; NSHigh, Not significantly different but higher than the check (Bio 9682).

**Table 2:** Output for the check variety Bio 9682 in the “yield vs. Mois”.

Name	Entry	Pedigree	Yield	Mois	Percentage yield	CodeMois
Bio 9682 (Check)	41	Bio 9682 (Check)	8.4	18.1	0	S
PM 21109L	30	Ped_30	9.7	17.9	15.0	S
R8050	35	Ped_35	9.6	18.1	14.2	NSHigh
PM 21111L	32	Ped_32	9.3	19.0	10.9	NSHigh
BIO 978	4	Ped_04	9.2	17.8	9.2	S
DKC 9226	9	Ped_09	9.1	18.3	7.9	NSHigh
ADV 7211	1	Ped_01	9.0	19.3	7.1	NSHigh

Mois, grain moisture content %; PercentageYield, yield above check (Bio 9682); CodeMois, Code for grain moisture content (%); S, similar and not significantly different; and NSHigh, not significantly different but higher than check (Bio 9682).

**Table 3:** Output for the check variety Bio 9682 in the “yield vs. HP”.

Name	Entry	Pedigree	Yield	HPs	Percentage yield	CodeHPs
Bio 9682 (Check)	41	Bio 9682 (Check)	8.4	36.7	0	S
PM 21109L	30	Ped_30	9.7	38.5	15.0	NSHigh
R8050	35	Ped_35	9.6	39.0	14.3	NSHigh
PM 21111L	32	Ped_32	9.3	38.6	10.9	NSHigh
BIO 978	4	Ped_04	9.2	38.8	9.2	NSHigh
DKC 9226	9	Ped_09	9.1	39.4	7.9	NSHigh
ADV 7211	1	Ped_01	9.0	36.0	7.1	S

HPs, Number of Harvested Plants, PercentageYield, yield above check (Bio 9682); CodeHPs, Code for number of harvested plants; S, similar and not significantly different; and NSHigh, not significantly different but higher than check (Bio 9682).

**Table 4:** Output for check Bio 9682 in the “yield vs AD vs Mois”.

Name	Entry	Pedigree	Yield	AD	Mois	Percentage yield	Code AD	Code Mois
Bio 9682 (Check)	41	Bio 9682 (Check)	8.4	55.0	18.1	0	S	S
PM 21109L	30	Ped_30	9.7	56.4	17.9	15.0	NSHigh	S
R8050	35	Ped_35	9.6	54.1	18.1	14.3	S	NSHigh
PM 21111L	32	Ped_32	9.3	54.2	19.0	10.9	S	NSHigh
BIO 978	4	Ped_04	9.2	54.5	17.8	9.2	S	S
DKC 9226	9	Ped_09	9.1	54.0	18.3	7.9	S	NSHigh

AD, Days to 50% anthesis; PercentageYield, Yield above the check (Bio 9682); CodeAD, Code for days to 50% anthesis; CodeMois, Code for grain moisture content (%); S, Similar, not significantly different; NSHigh, Not significant but higher than the check (Bio 9682).

**Table 5:** Output for the check variety Bio 9682 in the “yield vs. AD vs. HPs” Excel tab.

Name	Entry	Pedigree	Yield	AD	HPs	Percentage yield	Code AD	Code HPs
Bio 9682 (Check)	41	Bio 9682 (Check)	8.4	55.0	36.7	0	S	S
PM 21109L	30	Ped_30	9.7	56.5	38.5	15.0	NSHigh	NSHigh
R8050	35	Ped_35	9.6	54.1	39.0	14.3	S	NSHigh
PM 21111L	32	Ped_32	9.3	54.2	38.6	10.9	S	NSHigh
BIO 978	4	Ped_04	9.2	54.5	38.8	9.2	S	NSHigh
DKC 9226	9	Ped_09	9.1	54.0	39.4	7.9	S	NSHigh

AD, Days to 50% anthesis; HPs, Number of harvested plants; PercentageYield, Yield above the check (Bio 9682); CodeAD, Code for Days to 50% anthesis; CodeHPs, Code for number of harvested plants; S, Similar and not significantly different from the check (Bio 9682).

**Table 6:** Output for the check variety Bio 9682 in the “yield vs. Mois vs. HPs” .

Name	Entry	Pedigree	Yield	Mois	HPs	Percentage yield	Code Mois	Code HPs
Bio 9682 (Check)	41	Bio 9682 (Check)	8.4	18.1	36.7	0	S	S
PM 21109L	30	Ped_30	9.7	17.9	38.5	15.0	S	NSHigh
R8050	35	Ped_35	9.6	18.1	39.0	14.3	NSHigh	NSHigh
PM 21111L	32	Ped_32	9.3	19.0	38.6	10.9	NSHigh	NSHigh
BIO 978	4	Ped_04	9.2	17.8	38.8	9.2	S	NSHigh
DKC 9226	9	Ped_09	9.1	18.3	39.4	7.9	NSHigh	NSHigh
ADV 7211	1	Ped_01	9.0	19.3	36.0	7.1	NSHigh	S

Mois, Grain moisture content (%); HPs, Number of harvested plants; PercentageYield, Yield above the check (Bio 9682); CodeMois, Code for grain moisture content (%); CodeHPs, Code for number of harvested plants; S, Similar and not significantly different; NSHigh, Not significantly different but higher than the check (Bio 9682).

**Table 7:** Output for the check variety Bio 9682 in the “yield vs. AD vs. Mois vs. HPs” Excel tab.

Name	Entry	Pedigree	Yield	AD	Mois	HPs	Percentage yield	Code AD	CodeMois	Code HPs
Bio 9682 (Check)	41	Bio 9682 (Check)	8.4	55.0	18.1	36.7	0	S	S	S
PM 21109L	30	Ped_30	9.7	56.5	18.0	38.5	15.0	NS High	S	NS High
R8050	35	Ped_35	9.6	54.1	18.1	39.0	14.3	S	NS High	NS High
PM 21111L	32	Ped_32	9.3	54.2	19.0	38.6	10.9	S	NS High	NS High
BIO 978	4	Ped_04	9.2	54.5	17.8	38.8	9.2	S	S	NS High
DKC 9226	9	Ped_09	9.1	54.0	18.3	39.4	7.9	S	NS High	NS High

>AD, Days to 50% anthesis; Mois, Grain moisture content (%); HPs, Number of harvested plants; PercentageYield, Yield above the check (Bio 9682); CodeAD, Code for days to 50% anthesis; CodeMois, Code for grain moisture content (%); CodeHPs, Code for number of harvested plants; S, Similar and not significantly different; NSHigh, Not significantly different but higher than the check (BIO 9682).

Five experimental entries exceeded the predefined yield threshold while maintaining days to 50% anthesis statistically comparable to the check. PM 21109L (Entry 30) recorded the highest grain yield (9.7 t ha^-1^), representing a 15.0% advantage over Bio 9682, with an AD value of 56.5 days. R8050 (Entry 35) followed with a grain yield of 9.6 t ha^-1^ (14.3% advantage) and an AD value of 54.1 days. PM 21111L (Entry 32), BIO 978 (Entry 4), and DKC 9226 (Entry 9) exhibited yield advantages of 10.9%, 9.2%, and 7.8%, respectively, while maintaining AD values statistically comparable to the check.

According to the REMATTOOL-R classification criteria, the five selected entries did not differ significantly from Bio 9682 for days to 50% anthesis and may therefore be considered potential alternatives within the same maturity category. PM 21109L (Entry 30) exhibited the greatest yield advantage; however, its anthesis value was numerically higher than that of Bio 9682 despite remaining within the LSD threshold (CodeAD = NSHigh). Accordingly, although Entry 30 may be considered for advancement, interpretation should be made cautiously because repeated replacement using entries with slightly higher anthesis duration could gradually shift maturity classes over time. In contrast, entries 35, 32, 4, and 9 combined yield superiority with AD values equal to or lower than the check, representing more conservative alternatives for maintaining maturity-group integrity. Overall, REMATTOOL-R facilitates balanced selection by enabling simultaneous evaluation of grain yield and maturity-related traits rather than relying solely on yield superiority.

#### Yield versus grain moisture content (Yield vs. Mois)

**Table 2** presents entries exhibiting grain moisture content statistically comparable to Bio 9682 while achieving at least 5% higher grain yield. According to the REMATTOOL-R classification scheme, six entries (30, 35, 32, 4, 9, and 1) satisfied these criteria. PM 21109L (Entry 30) recorded the highest yield advantage (15.0%) while maintaining grain moisture comparable to Bio 9682. Entries 35, 32, 4, and 9 also demonstrated yield advantages ranging from 7.8% to 14.3% with moisture values statistically similar to the check. ADV 7211 (Entry 1) exhibited a 7.1% yield advantage but showed relatively higher grain moisture content, which may increase drying requirements and post-harvest management costs. Overall, several experimental entries combined superior yield performance with grain moisture profiles comparable to Bio 9682, indicating that final selection among candidate hybrids should consider breeding objectives, harvest management requirements, and other agronomic characteristics in addition to grain yield performance.

#### Yield versus number of harvested plants (yield vs. HP)

**Table 3** summarizes grain yield performance relative to harvested plant stand (HPs). Bio 9682 recorded a grain yield of 8.41 t ha^-1^ with an average harvested plant stand of 36.71 plants per plot. Six entries (30, 35, 32, 4, 9, and 1) achieved at least 5% higher grain yield than Bio 9682. PM 21109L (Entry 30), R8050 (Entry 35), PM 21111L (Entry 32), BIO 978 (Entry 4), and DKC 9226 (Entry 9) exhibited harvested plant stands ranging from 38.5 to 39.4 plants per plot together with yield advantages of 7.8–15.0%. These entries were classified as statistically similar or not significantly different but numerically higher than the check for harvested plant stand. ADV 7211 (Entry 1) achieved a grain yield of 9.01 t ha^-1^ with a harvested plant stand of 35.99 plants, remaining statistically comparable to Bio 9682. Overall, the results suggest that superior-performing entries generally combined higher grain yield with acceptable harvested plant stand levels. The integrated assessment provided by REMATTOOL-R assists in distinguishing genuine genetic yield improvement from performance differences arising from variation in plant establishment.

#### Yield versus anthesis days and grain moisture content (yield vs. AD vs. Mois)

**Table 4** presents experimental entries that achieved the required yield threshold while remaining statistically comparable to Bio 9682 for both days to 50% anthesis (AD) and grain moisture content at harvest. Five entries (30, 35, 32, 4, and 9) satisfied these criteria. PM 21109L (Entry 30) recorded the highest yield advantage over Bio 9682; however, although its AD value remained within the LSD threshold (CodeAD = NSHigh), its numerically higher anthesis value suggests that advancement should be interpreted cautiously because repeated replacement with such entries could gradually shift maturity duration within the maturity group. In contrast, entries 35, 32, 4, and 9 combined yield superiority with AD and grain moisture values comparable to or lower than those of the check, representing strong alternatives for maintaining relative maturity while improving productivity. Overall, these findings highlight the usefulness of REMATTOOL-R in identifying entries that simultaneously satisfy multiple maturity-related selection criteria rather than relying exclusively on grain yield.

#### Yield versus days to 50% anthesis and harvested plant stand (yield vs AD vs. HPs)

**Table 5** summarizes entries that were not significantly different from Bio 9682 for both days to 50% anthesis (AD) and harvested plant stand while exceeding the minimum yield threshold. Entries 30, 35, 32, 4, and 9 satisfied these criteria. PM 21109L (Entry 30) exhibited the highest grain yield advantage; however, its AD value was numerically greater than that of the check despite remaining statistically comparable (CodeAD = NSHigh). Consequently, its advancement should be interpreted cautiously in relation to long-term maturity maintenance. In contrast, entries 35, 32, 4, and 9 demonstrated substantial yield gains while maintaining days to 50% anthesis and harvested plant stand values comparable to Bio 9682, supporting their suitability as balanced candidates for advancement. Overall, these outputs enable simultaneous evaluation of yield performance together with important yield-influencing traits, thereby supporting more balanced advancement.

#### Yield versus grain moisture content and plant stand at harvest (yield vs. Mois vs. HPs)

**Table 6** includes entries that exceeded Bio 9682 by at least 5% in grain yield while remaining statistically comparable to the check for both grain moisture content and harvested plant stand. Five principal entries (30, 35, 32, 4, and 9) fulfilled these criteria. PM 21109L (Entry 30) recorded the largest yield advantage, followed by entries 35, 32, 4, and 9. ADV 7211 (Entry 1), despite exhibiting favorable performance for some individual traits, did not satisfy all combined selection criteria simultaneously and therefore requires more cautious interpretation within the multi-trait framework. The combined evaluation of grain moisture content and harvested plant stand provides additional insight into whether yield superiority is associated with acceptable maturity behaviour and fair stand establishment, thereby improving confidence in identifying candidates showing genuine agronomic improvement relative to the check hybrid.

#### Yield versus days to 50% anthesis, grain moisture content, and plant stand at harvest (yield vs. AD vs. Mois vs. HPs) option

**Table 7** presents entries that simultaneously satisfied all REMATTOOL-R selection criteria: grain yield superiority (≥5% above Bio 9682) together with no significant differences from the check for days to 50% anthesis (AD), grain moisture content (Mois), and harvested plant stand (HPs). Five entries (30, 35, 32, 4, and 9) met these stringent multi-trait requirements. PM 21109L (Entry 30) recorded the highest yield advantage among the selected entries; however, because its AD value was numerically higher than that of Bio 9682 despite remaining statistically comparable (CodeAD = NSHigh), its advancement should be interpreted cautiously in the context of maturity maintenance. In contrast, entries 35, 32, 4, and 9 achieved yield superiority while maintaining maturity-related and harvested plant stand characteristics comparable to the check, representing strong candidates for advancement under a conservative maturity framework.

ADV 7211 (Entry 1) was not retained in **Table 7** despite favorable performance in certain individual comparisons. Although the entry satisfied the minimum yield threshold and performed adequately for harvested plant stand and grain moisture content in some individual analyses, it did not simultaneously satisfy all maturity- and adaptation-related criteria required under the REMATTOOL-R multi-trait filtering approach. Its exclusion therefore reflects the strict combined-trait selection framework rather than poor performance for any single characteristic. Overall, these results demonstrate the value of REMATTOOL-R for multi-trait decision support by enabling simultaneous interpretation of yield superiority together with maturity- and stand-related traits within a unified selection framework.

### Validation of REMATTOOL-R selections using AICRP promotion outcomes

The superior hybrids identified through REMATTOOL-R were compared with official advancement decisions under the AICRP maize evaluation system (**Table 8**). Among the five entries selected by REMATTOOL-R under the integrated multi-trait framework (PM21109L, R8050, PM21111L, BIO978, and DKC9226), all five were promoted from NIVT to AVT-I under the official AICRP advancement framework. Furthermore, R8050, PM21111L, and DKC9226 continued to be advanced from AVT-I to AVT-II during subsequent evaluation cycles, indicating sustained agronomic merit across testing stages.

**Table 8:** Comparison of REMATTOOL-R identified entries with official AICRP promotion outcomes.

Hybrid	REMATTOOL Selected	NIVT→AVT-I (AICRP)	AVT-I→AVT-II (AICRP)
PM 21109L	Yes	Yes	No
R8050	Yes	Yes	Yes
PM 21111L	Yes	Yes	Yes
BIO978	Yes	Yes	No
DKC 9226	Yes	Yes	Yes

In contrast, PM21109L and BIO978, although promoted from NIVT to AVT-I, did not progress further to AVT-II, indicating that additional evaluation criteria, including pest and disease response, fertilizer and spacing studies, and breeder assessments, may influence final advancement decisions beyond the core REMATTOOL-R framework.

This agreement between REMATTOOL-R outputs and sequential multi-stage AICRP promotion decisions, particularly during early testing stages where large numbers of entries are evaluated, supports the practical reliability of the tool for identifying hybrids possessing high yield potential together with key selection criteria, including desirable days to 50% anthesis, grain moisture content, and harvested plant stand.

**Supplementary Table 1** and **S2** present the promotion status of late-maturity maize hybrids evaluated in TR.947 (NIVT to AVT-I) and TR.1052 (AVT-I to AVT-II), respectively, including mean grain yield, yield superiority over the check, maturity parameters, disease reactions, and final breeding recommendations under CWZ conditions.

## Discussion

The present study evaluated the usefulness of REMATTOOL-R for validating and streamlining the identification of superior maize hybrids under the All India Coordinated Research Project (AICRP) maize testing framework. Under the conventional AICRP evaluation system, hybrids are assessed across multiple agro-climatic zones and advanced sequentially from National Initial Varietal Trials (NIVT) to Advanced Varietal Trials (AVT-I and AVT-II) using criteria including grain yield superiority, maturity behaviour, disease resistance, adaptation, and statistical performance relative to commercial checks (AICRP-Maize, 2023). Although this system is scientifically robust, interpretation of multi-location datasets involving numerous agronomic and maturity-related traits can be laborious, time-intensive, and occasionally susceptible to computational inconsistencies or subjective interpretation, potentially leading to under-recognition of promising hybrids.

The present investigation demonstrated that REMATTOOL-R effectively simplified hybrid discrimination by integrating grain yield with biologically important maturity- and adaptation-related traits within a single analytical framework. Using a limited set of key traits—grain yield, days to 50% anthesis, grain moisture content, and harvested plant stand—the analytical outputs identified hybrids largely consistent with those prioritized under conventional AICRP advancement procedures. Importantly, all five hybrids selected through the REMATTOOL-R integrated multi-trait framework (PM21109L, R8050, PM21111L, BIO978, and DKC9226) corresponded with official NIVT-to-AVT-I promotion decisions under the AICRP maize testing system. Furthermore, R8050, PM21111L, and DKC9226 progressed further from AVT-I to AVT-II during subsequent evaluation cycles, indicating sustained agronomic merit across advancement stages. This progression across sequential testing stages provides an independent external validation of the REMATTOOL-R selection framework under operational breeding programme conditions. This concordance between REMATTOOL-R outputs and sequential breeder-led promotion outcomes supports both the reliability of the existing AICRP selection framework and the practical utility of REMATTOOL-R as a breeder-oriented decision-support approach (Machida et al., 2023).

Grain yield remains the principal criterion for hybrid advancement in maize breeding programmes. In the present study, several entries exhibited clear yield superiority over the commercial check while maintaining acceptable maturity and adaptation characteristics. Comparable gains in yield performance and adaptation efficiency have been reported in recent multi-location maize breeding studies emphasizing integrated selection approaches (Assefa et al., 2021; Cairns et al., 2022; Worku et al., 2023). Within the AICRP framework, superior hybrids are generally identified separately for different testing zones. However, identification of hybrids exhibiting broader adaptation across multiple zones could simplify seed production logistics and improve deployment efficiency for both public and private breeding programmes.

Days to 50% anthesis and grain moisture content are important maturity-related traits influencing hybrid advancement, farmer acceptance, and post-harvest management. Delayed flowering and elevated grain moisture are generally undesirable because they may prolong crop duration and increase drying costs. In the present study, graphical visualization of grain yield in relation to anthesis duration and grain moisture facilitated rapid identification of entries combining high productivity with acceptable maturity behaviour. The positive association between anthesis duration and grain moisture observed in the correlation analysis agrees with reports indicating that later-flowering hybrids frequently retain higher grain moisture because of prolonged physiological maturity (Li et al., 2021). Similar relationships between flowering behaviour and grain dehydration dynamics have been documented in recent maize physiological studies (Yang et al., 2025).

An important outcome of the study was the identification of hybrids combining superior grain yield with maturity characteristics statistically comparable to the commercial check. Such entries represent meaningful genetic improvement without major shifts in maturity duration. Nevertheless, the present findings also highlight the importance of cautious interpretation when high-yielding entries exhibit numerically greater anthesis duration than the check despite remaining statistically comparable. Repeated advancement of such entries may gradually alter maturity categories over successive varietal replacement cycles. This consideration is particularly relevant under intensive and multi-cropping production systems where maturity duration strongly influences cropping schedules and hybrid acceptability.

The positive relationship between harvested plant stand and grain yield emphasizes the importance of adequate crop establishment in maximizing hybrid productivity. However, superior entries retained yield advantages after accounting for differences in harvested plant stand, suggesting that their performance was unlikely to be explained solely by stand establishment differences, although plant stand effects should still be considered during interpretation. Similar relationships among plant density, crop stand, and grain yield stability have been reported in modern maize hybrids evaluated across diverse production environments (Testa et al., 2021; Lindsey and Thomison, 2022; Farnham, 2022). These observations support inclusion of harvested plant stand as an important component of integrated hybrid assessment.

Another practical advantage of REMATTOOL-R was the generation of easily interpretable graphical outputs together with interactive correlation analyses. The x–y plots, density distributions, and correlation matrices facilitated rapid interpretation of relationships among grain yield, anthesis duration, grain moisture, and harvested plant stand. Trait association analyses are widely used in maize improvement programmes to understand direct and indirect relationships influencing hybrid performance, and similar applications have been reported in recent maize breeding studies (Kumar et al., 2021; Bartaula et al., 2021; Nzuve et al., 2022).

Multi-environment evaluation is fundamental for identifying stable and widely adapted maize hybrids suitable for coordinated testing programmes. Analytical approaches such as AMMI (Additive Main Effects and Multiplicative Interaction) and GGE biplot analysis are widely used for investigating genotype × environment (G × E) interactions, stability patterns, and adaptation across testing locations. These methods provide detailed information on genotype performance, mega-environment delineation, and specific versus broad adaptation; however, interpretation of their outputs often requires specialized statistical expertise and familiarity with multivariate analyses (Gauch, 2006; Yan and Tinker, 2006; Yan, 2014).

Compared with these approaches, REMATTOOL-R is designed primarily as a breeder-oriented decision-support tool emphasizing practical advancement decisions through simultaneous interpretation of grain yield, maturity-related traits, and harvested plant stand. Unlike conventional breeder selection based largely on extensive statistical tables or independent trait comparisons, REMATTOOL-R integrates biologically relevant traits into intuitive graphical and tabulated outputs that can be interpreted rapidly by breeders, evaluation committees, and decision makers. Thus, REMATTOOL-R should be viewed as complementary to, rather than a replacement for, established multi-environment analytical approaches such as AMMI and GGE biplot analyses. While AMMI and GGE provide deeper insight into genotype × environment interactions and stability patterns, REMATTOOL-R offers a simplified framework for rapid multi-trait hybrid discrimination and maturity-aware identification of superior hybrids meeting predefined selection criteria.

Because REMATTOOL-R identified hybrids that closely matched official AICRP advancement outcomes during early testing stages, the framework may help breeders prioritize promising entries more efficiently within large coordinated evaluation pipelines. Such early-stage filtering could potentially reduce evaluation burden, improve utilization of testing resources, and accelerate advancement decisions in maize breeding programmes.

Despite its practical advantages, several methodological limitations should be acknowledged. First, the analytical outputs are dependent on the quality of the underlying least-square means generated from experimental datasets. Inaccurate field observations, inconsistent trial management, or unreliable trait estimates may influence the resulting classifications and visual interpretations.

Second, performance of the approach may be affected by environmental imbalance and missing observations, which are common challenges in multi-environment trials. The framework also assumes biologically consistent relative ordering of check hybrids for maturity-related traits; deviations from expected check behaviour may indicate unreliable flowering or grain moisture observations and may reduce confidence in maturity-based interpretation. Unequal representation of testing locations, failed trials, or incomplete trait measurements can influence trait estimates and may reduce confidence in comparisons among entries. Similarly, sparse datasets containing limited environmental representation or small numbers of valid observations may constrain the robustness of maturity and yield interpretations.

While the present implementation focused on the four core REMATTOOL-R traits (grain yield, days to 50% anthesis, grain moisture content, and harvested plant stand), official AICRP advancement decisions additionally consider disease resistance, insect–pest resistance, and breeder evaluations. The promotion outcomes examined in the present study indicate that such factors may contribute to final advancement decisions beyond the core REMATTOOL-R framework. Therefore, future development of the tool could explore integration of additional biotic stress and breeder assessment parameters to further strengthen alignment with coordinated varietal evaluation systems.

Nevertheless, the present findings demonstrate that REMATTOOL-R provides an efficient and transparent framework for integrating yield superiority with maturity-related and stand-related traits, thereby supporting rapid advancement decisions within coordinated breeding systems.

An additional strength of the REMATTOOL-R framework lies in its computational accessibility and reproducibility. The tool is available through the CIMMYT Dataverse repository together with supporting files and user guidance, facilitating broader adoption by breeding programmes and researchers. Standardized graphical outputs, automated tabulation, and transparent classification criteria reduce manual calculations and improve reproducibility in advancement decisions.

Future improvements could further strengthen the analytical framework. Potential extensions include sensitivity analyses evaluating the influence of varying yield thresholds, validation across multiple years and testing cycles, integration of genotype stability indices, and comparative benchmarking against AMMI, GGE biplot, and related multi-environment analytical methods. Such developments would broaden the applicability of REMATTOOL-R and enhance its utility as a complementary tool for objective hybrid evaluation and selection.

## Conclusion

The present study demonstrated the usefulness of REMATTOOL-R as a reliable decision-support tool for validating the AICRP maize hybrid promotion framework. By integrating grain yield with important maturity- and adaptation-related traits, including days to 50% anthesis, grain moisture at harvest, and harvested plant stand, the tool enabled rapid, transparent, and objective identification of superior hybrids from multi-location trial data.

Five experimental entries—PM 21109L (30), R8050 (35), PM 21111L (32), BIO 978 (4), and DKC 9226 (9)—exhibited more than 5% grain yield superiority over the standard check Bio 9682 while maintaining maturity and plant stand characteristics that were statistically comparable to the check. Although Entry 30 recorded the highest yield advantage, careful consideration of maturity-related traits remains important to avoid unintended shifts in maturity duration during varietal replacement.

Importantly, all five hybrids identified through REMATTOOL-R corresponded with official NIVT-to-AVT-I advancement decisions within the AICRP maize testing framework. Moreover, three selected hybrids (R8050, PM21111L, and DKC9226) progressed further to AVT-II during subsequent testing cycles, providing independent validation of the REMATTOOL-R analytical framework under real breeding programme conditions.

Overall, the findings demonstrate that REMATTOOL-R provides a transparent, reproducible, and practically relevant complementary framework for objective hybrid advancement decisions in coordinated maize breeding programmes. The framework supports efficient multi-trait evaluation and prioritization of experimental entries and offers a practical approach for improving transparency, consistency, and decision support within breeder-oriented hybrid evaluation systems.

## Data Availability

The original contributions presented in the study are included in the article/**Supplementary Material**. Further inquiries can be directed to the corresponding author.
