# Supplementary material for: Validating superior maize hybrids in all India coordinated trials using REMATTOOL-R: a decision support approach
Source: Front Plant Sci. 2026 Jul 7;17:1885434. doi: 10.3389/fpls.2026.1885434 (PMC13385124; doi:10.3389/fpls.2026.1885434)
Supplement: Supplementary file 1 [file Table1.docx]

**Supplementary Table S1. CWZ- Promotion list (Late) entries from TR. 947 NIVT to AVT I.**

| **Hybrids name** | **Mean Grain Yield (t/ha)** | **Rank** | **% Superiority** | **Days to 75% dry husk** | **Days to Silking** | **Days to Anthesis** | **Remarks_Breeding** | **Disease score and reaction** | **Final Remarks** |
| --- | --- | --- | --- | --- | --- | --- | --- | --- | --- |
| R8050 | 9.570 | 1 | 14.7 | 92 | 56 | 54 | Promoted | CLS(3.4), Cyst/Plant(23.8, S), FSR(3.9), RDM(12.7) | Promoted |
| PM 21109L | 9.537 | 2 | 14.3 | 94 | 59 | 56 | Promoted | CLS(4.0), Cyst/Plant(11.0, S), FSR(1.5), RDM(18.1) | Promoted |
| PM 21111L | 9.297 | 3 | 11.4 | 94 | 57 | 54 | Promoted | CLS(5.0), Cyst/Plant(16.8, S), FSR(5.9), RDM(3.8) | Promoted |
| BIO 978 | 9.124 | 4 | 9.3 | 94 | 57 | 54 | Promoted | CLS(4.0), Cyst/Plant(10.5, S), FSR(6.1), RDM(23.6) | Promoted |
| DKC 9226 | 8.971 | 5 | 7.5 | 93 | 57 | 54 | Promoted | CLS(2.9), Cyst/Plant(33.0, S), FSR(3.6), RDM(24.6) | Promoted |
| ADV 7211 | 8.937 | 6 | 7.1 | 97 | 60 | 58 | Promoted | CLS(4.2), Cyst/Plant(22.3, S), FSR(4.0), RDM(5.6) | Promoted |
| PM 21108L | 8.917 | 7 | 6.8 | 93 | 59 | 56 | Promoted | CLS(4.4), Cyst/Plant(21.5, S), FSR(4.6), RDM(18.7) | Promoted |
| PM 21107L | 8.915 | 8 | 6.8 | 95 | 58 | 55 | Promoted | CLS(1.7), Cyst/Plant(30.2, S), FSR(4.3), RDM(19.6) | Promoted |
| Bio 9682 (Check) | 8.346 | 17 | 0.0 | 95 | 57 | 55 |  | CLS(4.3), Cyst/Plant(22.8, S), FSR(3.6), RDM(11.2) |  |
| CD (5%) | 0.842 |  |  |  |  |  |  |  |  |
| Cut off Yield | 8.728 |  |  |  |  |  |  |  |  |

**Abbreviations:** CWZ, Central Western Zone; NIVT, National Initial Varietal Trial; AVT, Advanced Varietal Trial; CLS, Curvularia Leaf Spot; FSR, Fusarium Stalk Rot; RDM, Rajasthan Downy Mildew; MCN, Maize Cyst Nematode; S, Susceptible; MR, Moderately Resistant.

**Supplementary Table S2. CWZ- Promotion list (Late) entries from TR. 1052 AVT I to AVT II Late CWZ.**

| **Hybrids name** | **Mean Grain Yield (t/ha)** | **Rank** | **% Superiority** | **Days to 75% dry husk** | **Days to Silking** | **Days to Anthesis** | **Imp disease-FSR, MCN (on the basis of national average)** | **Remarks Plant Pathology** | **Entomology data** | **Remarks Breeding** | **Final Remarks** |
| --- | --- | --- | --- | --- | --- | --- | --- | --- | --- | --- | --- |
| R8050 | 8.501 | 1 | 16.5 | 89.95 | 57.81 | 54.95 | FSR (4.3, MR), MCN (27.9, S) | Promoted | 3.57 MR | Promoted | Promoted |
| PM 21111L | 7.925 | 2 | 8.6 | 90.67 | 58.19 | 55.52 | FSR (4.8, MR), MCN (18.6, S) | Promoted | 4.05 MR | Promoted | Promoted |
| DKC 9226 | 7.914 | 3 | 8.5 | 90.29 | 57.43 | 54.43 | FSR (3.9, MR), MCN (16.8, S) | Promoted | 3.11 MR | Promoted | Promoted |
| Bio 9682 (Check) | 7.295 | 5 | 0.0 | 90.57 | 58.05 | 55.24 | FSR (4.9, MR), MCN (19.6, S) |  | 3.97 MR | Check |  |
| Z4CD (5%) | 0.597 |  |  |  |  |  |  |  |  |  |  |
| Cut off Yield | 7.904 |  |  |  |  |  |  |  |  |  |  |

**Abbreviations:** CWZ, Central Western Zone; NIVT, National Initial Varietal Trial; AVT, Advanced Varietal Trial; CLS, Curvularia Leaf Spot; FSR, Fusarium Stalk Rot; RDM, Rajasthan Downy Mildew; MCN, Maize Cyst Nematode; S, Susceptible; MR, Moderately Resistant
